# Patient, Care Partner, and Health Care Professional Opinion of the Lecanemab Autoinjector for Subcutaneous Delivery in Early Alzheimer’s Disease Patients

**DOI:** 10.1002/alz70861_108809

**Published:** 2025-12-23

**Authors:** Katie Jamison, Leah Zullig, R. Scott Turner, Daryl Jones

**Affiliations:** ^1^ Eisai Inc., Nutley, NJ USA; ^2^ Duke University School of Medicine, Durham, NC USA; ^3^ Georgetown, University, Washington DC, DC USA

## Abstract

**Background:**

Lecanemab‐irmb (LEQEMBI^®^) is an amyloid‐targeting monoclonal antibody indicated for the treatment of patients with AD in the mild cognitive impairment (MCI) or mild AD dementia stage (early AD). Lecanemab is approved by several countries as an intravenous formulation requiring bi‐weekly administration; and in the USA patients may transition to monthly infusions after 18 months of treatment. There is a subcutaneous formulation of lecanemab in development that will be administered via an autoinjector, which may lead to improved treatment experience. In human factors studies, the autoinjector has demonstrated safe and effective use for intended users, intended uses, and intended use environments. The objective of this study is to examine patient, care partner, and health care professional (HCP) opinion of the lecanemab autoinjector.

**Method:**

The Pilot (N = 24) and Main Study (N = 278) included participants from three groups: patients with early AD; care partners for adults with early AD; and HCPs (nurses, nurse practitioners, and physician assistants). In‐person study visits included device familiarization and several measures of acceptability and feasibility, including the Self‐Injection Assessment Questionnaire (SIAQ), Acceptability of Intervention Measure, and Feasibility of Intervention Measure. Interim Pilot results (cut‐off date: April 18, 2025) are reported here.

**Result:**

In the interim analysis, 18 participants had complete datasets (2 patients, 8 care partners, 8 HCPs). On the SIAQ, 100% of users reported that it was “easy” or “very easy” to use the autoinjector; 100%, 75%, and 88% of patients, care partners, and HCPs selected “very easy,” respectively. 100% of users reported that they were “satisfied” or “very satisfied” with this way of taking medication; 50%, 75%, and 88% of patients, care partners, and HCPs selected “very satisfied,” respectively. Finally, 100% of users reported that they were “very” or “extremely” confident about giving an injection in the right way; 100%, 75%, and 100% of patients, care partners, and HCPs selected “extremely” confident, respectively.

**Conclusion:**

Patients, care partners, and HCPs found the autoinjector easy to use, expressing high levels of satisfaction and confidence (interim data). The full analysis will further describe ease of use, satisfaction, and confidence with the lecanemab autoinjector.

